# Twenty-five years of Alabama’s Rural Health Leaders Pipeline: what difference has it made?

**DOI:** 10.3389/fmed.2025.1555987

**Published:** 2025-06-24

**Authors:** John R. Wheat

**Affiliations:** Department of Family, Internal and Rural Medicine, College of Community Health Sciences, University of Alabama, Tuscaloosa, AL, United States

**Keywords:** rural physicians, medical education, pipeline, educational model, minority populations, cultural dissonance, evaluation research

## Abstract

**Introduction:**

This community case report describes the Alabama Rural Health Leaders Pipeline operated as a demonstration research project, 1993–2017, tests of its effectiveness, and supportive studies. The purpose was to demonstrate production of physicians for Alabama’s diverse rural population. The community-centric conceptual model was operationalized as two precollege summer pipeline programs and a master’s in rural community health/rural medicine track that engaged 1,045 rural Alabama students over 25 years: 651 Rural Health Scholars after 11th grade, 174 Rural Minority Health Scholars after the 12th, and 220 Rural Medical Scholars in the combined MS/MD track. Rural students, rural community-based instruction, family medicine instructors, and community engagement were key components.

**Method:**

Review of Rural Health Leaders Pipeline publications. Four papers evaluated medical student academic performance, specialty choice, geographic location of practice, and production of other health professionals. Sixteen explored (a) factors associated with limited physician distribution in the Black Belt and (b) circumstances that engaged institutional and community collaborators in program development.

**Findings:**

Compared to peers in traditional medical education, rural medical track alumni more frequently chose family medicine specialty (*p* < 0.001, OR = 15.6) and rural Alabama practice (*p* < 0.001, OR = 6.4) with no difference in academic performance (*p* > 0.05). Few rural medical track alumni established practice in the Black Belt, with many hypothetical factors identified. RHLP also produced other health professionals. Contextual studies engaged local physicians, institutional colleagues, school systems, the agricultural community, and health care entities in planning, collaboration, and advocacy regarding rural adaptations of admissions, curriculum, pedagogy, and educational context.

**Discussion:**

The demonstration proved successful across much of rural Alabama, gained continuing state funding, and was institutionalized and expanded in the University of Alabama System. Further expansion is required to meet rural needs. Limited impact in the Black Belt remains a challenge for rural medical education and provides opportunities for future research.

## Introduction

Worldwide, medical education that assures personal physicians for rural populations progresses slowly. The Rural Health Leaders Pipeline (RHLP), a rural medical education (RME) initiative in Alabama, was a demonstration research project from 1993–2017 in response to different philosophies propelling medical education. The traditional model linking universities, hospitals, urban populations, and laboratory research stimulated an oversupply of urban specialists and too few primary care physicians. Popular demand for personal medical care brought forth in the 1960s the specialty of Family Medicine and federal support for care of poor and elderly populations (1, 2). However, traditional medical schools in poor states, such as Alabama, were concerned that the costs of medical education differentiated to produce family physicians would divert funds from existing operations. The question arose-could a medical school simultaneously advance scientific discovery and service population healthcare needs.

In 1972, the University of Alabama (UA) attracted Bill Willard, who had led the American Medical Association’s efforts creating the specialty of Family Medicine (2), to build the College of Community Health Sciences (CCHS) in Tuscaloosa to produce family physicians for Alabama (3), a rural state. Tuscaloosa is located at the nexus of rural Appalachian and Black Belt regions, both with chronic doctor shortages. The established medical school (SOM) in Birmingham, Alabama’s largest city, had a growing reputation for research and specialty care (4). Limited state funding pushed the research and servicephilosophies into competition, which delayed the completion of Willard’s plan. CCHS established an independent family practice residency, while its Doctor of Medicine (MD) program evolved as a regional extension of SOM (5).

The 1980s economic downturn compounded the shortage of rural physicians. Alabama declared a rural health crisis in 1989 and created an agency with appropriations to enhance production of rural family physicians (6). This mandate united SOM and CCHS to address RME. They recruited the author for this purpose in 1990 (5). Tensions between the two camps remained, but each accepted the demonstration research strategy to determine the utility of a long-term commitment to RME.

By 1990, RME programs in Minnesota (7), Pennsylvania (8), and Illinois (9) were reporting development of comprehensive programs linking rural students, family medicine instruction, and community clerkships. These programs were taken as benchmarks for planning Alabama’s RME initiative.

A fundamental question was what modifications to the benchmark model would be necessary for success in Alabama. CCHS had the family practice residency and the SOM clinical branch campus with a rotation in rural family practice and community medicine, but few rural students were admitted to SOM. Rural students’ premedical preparation and support during the preclinical years at Birmingham were foremost concerns when populations of Alabama and benchmark states were compared. Alabama had a lower education level, greater poverty, more rural, more diversity, poorer health ranking, and lower doctor to population ratio (10).

Alabama’s history inscribes exploitation of natural and human resources (i.e., fertile soil, timber, minerals, slavery, sharecropping or tenant farming, mining, and institutional racism) (4, 11, 12). A diverse population (i.e., 66% White, 27% Black, and 5% Latinx) distributes among 67 counties of which 55 are rural with persistent health care shortages. A region of 17 rural counties with dark soil, the Black Belt, produced the historical cotton economy and continues to maintain predominantly African American (AA) communities living with poverty and associated determinants of health, including below average educational offerings (12). The Black Belt represents one of America’s geographical subpopulations with severe need of physicians (i. e., black, non-metropolitan and low income, South) (13). Table 1 contrasts population and physician supply among the Black Belt, the State of Alabama, and the US. The disparity in the Black Belt (i.e., one physician per 3,500 population vs. one per 1,300 nationally) forecasts efforts required to produce and maintain physicians in this region.

**Table 1:** Alabama’s Black Belt region by population, African American percentage, and PCP supply, 2020.

Region	Population (73)	Black or AA % (73)	Avg. population per PCP^a^ (74)
Black Belt (17 counties) (75)	558,473	60	3593
Alabama (all 67 counties)	5,024,356	27	1540
United States	329,500,000	12	1330

PCP, primary care physician. Data were obtained from the University of Alabama Center for Economic Development (75), the US Census (73), and the University of Wisconsin Population Health Institute (74). Reprinted from Wheat et al. (26). Used by permission under CCBY-NC-ND 4.0.

a Montgomery County in the Black Belt was excluded in this column because it includes the capitol city and has a ratio of 1030 population per PCP.

Before RHLP, Alabama’s higher education officials discounted rural students’ preparation and “fit” for medical school. That schools in the Black Belt were either all black public or all white private was another complicating factor (12). Rural communities, however, insisted on “growing our own” physicians who identified with the local population, prompting a vision of the Rural Health Leaders Pipeline (RHLP). Healthcare entities, farmer groups, and community leaders were motivated to help create a predictable supply of rural family physicians (14).

Rural students approached medical careers, typically, through hometown schools, in-state colleges, and Alabama’s two public medical schools. Endorsing the communities’ proposition of local students, RHLP was conceived to attract rural students to healthcare careers and nurture their ability and resolve to become rural family doctors and leaders in developing healthy communities in Alabama. Students were considered rural if from a rural county or a town of less than 2500 (15).

## Details—program design and research methods

### Purpose

This paper describes the RHLP, including its conceptual model, operationalization, evaluation, and associated research, and forecasts future developments.

### Reporting strategy

The report is a community case-report limited to and reflecting research associated with RHLP. The author selected peer-reviewed research articles indexed by PubMed, categorized them according to purpose, synthesized the findings, and discussed future steps. The UA Institutional Review Board approved the research reported in each article.

### Conceptual model

**Figure 1:**
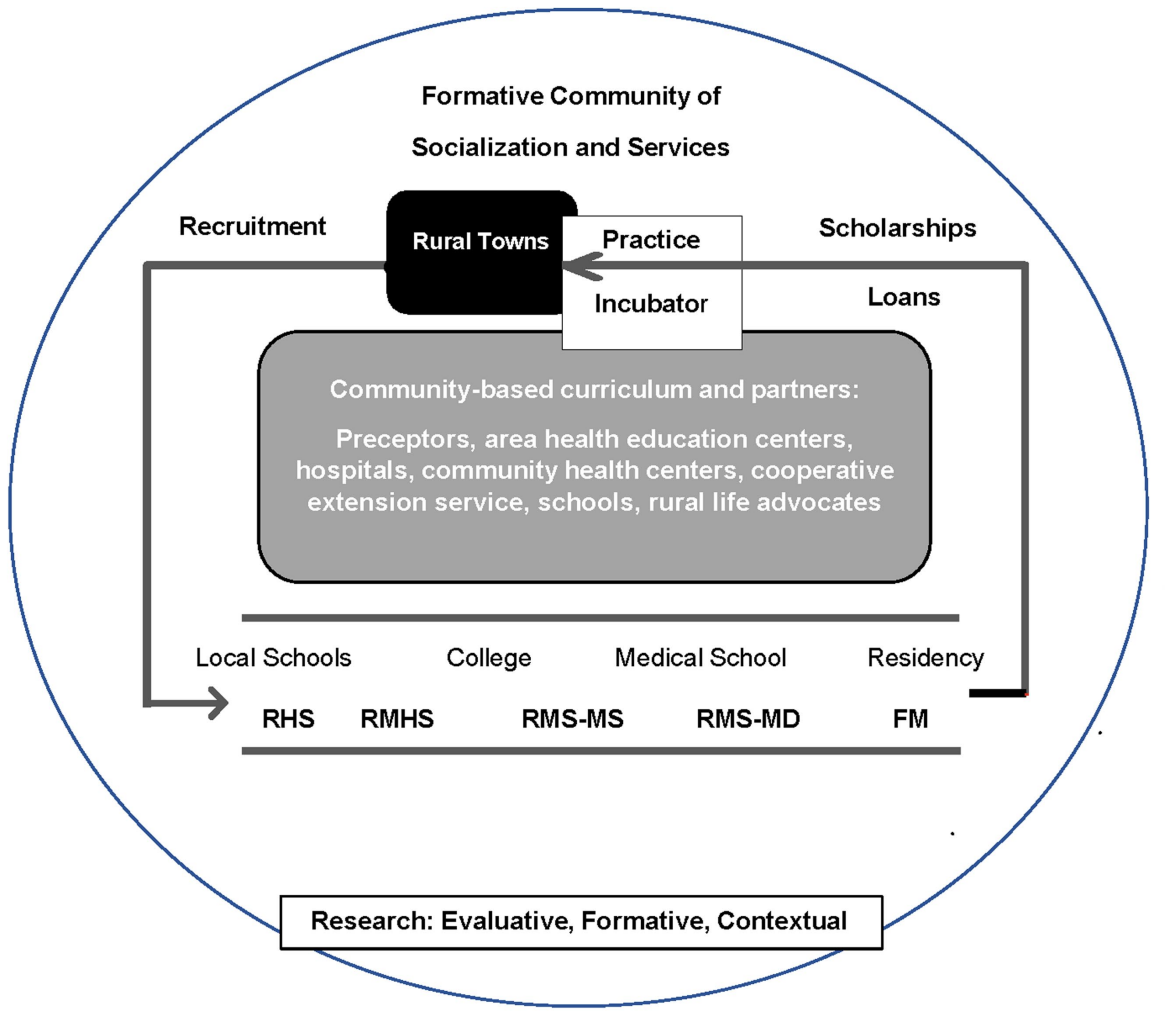
Rural Health Leaders Pipeline model to produce rural physicians. RHS, Rural Health scholars; RMHS, Rural minority health scholars; RMS-MS, Rural medical scholars-master of science; RMS-MD, Rural medical scholar-medical doctor; FM, Family Medicine. Adapted in part from National Commission on Community Health Services (1) and Wheat et al. (15).

The RHLP model conceptualizes a way to produce physicians for rural Alabama and to guide its evaluation. We summarized previously literature that informed construction of the model (16) and consulted seminal work of Willard (2, 3, 17) and benchmark programs (8, 18) to draft the RME plan that was authenticated by rural physicians, hospital administrators, and community leaders (14). The resulting model, depicted in Figure 1, depended on rural students, their formative communities (19), family medicine instructors, and a longitudinal community-based curriculum (7–9) starting in high school. The influence of a student’s formative community is included throughout the model (15) including the local community in which they were socialized and the larger community with agencies and institutions on which their families depend for services (1). The recommendation to complete four years of medical school at the branch campus was not accomplished, maintaining instruction in the preclinical medical sciences at the main campus at the periphery of rural students’ formative communities.

The model locates RME within students’ formative community, which is hypothesized to be a decisive factor in the development of rural physicians. It provides an identity and significant others (e.g., family, peers, and community members) and agencies (e.g., school, health care, cooperative extension, and church) that encourage students, affirm their aspirations, and nurture their development. Students learn from local patients and instructors whose practices are informed by local needs.

### Operational model

The demonstrated RHLP included 3 educational program components. Two programs supplemented students’ local education with summer pipeline programs at UA. The other formed an RME track. Each program was initiated with input and collaboration with rural stakeholders. Program emphasis was supported or adjusted by information generated through these collaborations.

The Rural Health Scholars Program (RHS) (15), 1993–2017, enrolled 642 rural 11th grade students in a five-week on-campus summer program that was informed by UA’s experience with college preparatory programs (20). We introduced students to college life, health careers, and rural healthcare advocates through chemistry and creative writing courses (6 semester hours), peer group activities, field trips, and seminars with rural college students, health professionals, and advocates.

The Rural Medical Scholars Program (RMS) (16), 1996–2017, engaged 230 rural students to promote progression toward rural family practice. In-state colleges were targeted for recruitment (21). The five-year RME track included a one-year master’s program (RMS-MS) focused on rural community health supplemented with advanced biomedical science courses followed by medical school (RMS-MD) with preclinical sciences at SOM main campus and clinical medicine taught from the regional campus emphasizing rural community-based instruction. Program specifications addressed admissions, campus, curriculum, community engagement, and family physician instructors (15, 16).

The Rural Minority Health Scholars Program (RMHS) (22, 23), 2000–2017, involved 179 students in response to progress reviews showing that students from the Black Belt were well represented among RHS but their admission to the RMS program and medical school lagged. Ten RMHS per year were accepted from rural minority applicants after completing high school; many had been RHS. Conducted in parallel with the RHS, RMHS took courses in premedical sciences and seminars on social determinants of health. Table 2 summarizes participation in RHLP programs.

**Table 2:** RHLP participants by program and race, 1993–2017.

	No. and % participants		
	African American	Non-African American	Total
RHLP programs	*N* (%)	*N* (%)	*N* (%)
Post-11th grade program (RHS), 1993–2017	195 (30.0)	456 (70.0)	651 (100)
Post-12th grade program (MRHS), 2000–2017	169 (97.1)	5 (2.9)	174 (100)
MS/MD track (RMS), 1996–2017	16 (7.3)	204 (92.7)	220 (100)
Total	380 (36.4)	665 (63.6)	1,045 (100)

MRHS, Minority Rural Health Scholars; MS/MD, Master of Science/Doctor of Medicine; RHLP, Rural Health Leaders Pipeline; RHS, Rural Health Scholars; RMS, Rural Medical Scholars. Adapted from Wheat et al. (26). Used by permission under CCBY-NC-ND 4.0.

Students could be admitted into RHLP at any one of the component programs but had to apply separately for each one. Investigators recruited students directly at local schools (RHS and RMHS) or colleges (RMS) and indirectly through groups representing educators, health professionals, hospital administrators, farmers, and government officials. The admissions committee for RHLP programs included members from rural Alabama, faculty, and program personnel. Admission criteria included attendance of a rural school, recommendations by community leaders, and scholastic performance. SOM interviewed RMS applicants with at least eight years of rural Alabama residence, GPA ≥ 3.2 (out of 4), and Medical College Admission Test (MCAT) score ≥24 old or 495 new.

### RHLP diversity climate

Program staff had rural backgrounds and represented Alabama’s population subsets. The medical director served as a continuous role model throughout all component programs while maintaining a rural family practice in a nearby small town. Programs included experiences in or near students’ home communities and interactions with individuals who supported their rural ambition. Family members participated in students’ community assessments and program ceremonies.

### RHLP research

Table 3 shows the distribution of 20 research articles among three categories: I. Evaluation (4), II. Formative (2), and III. Contextual (14) studies and characterized them by research questions and design.

**Table 3:** Rural Health Leaders Pipeline (RHLP) research, 1993–2017.

Research question	Research design
I. Evaluation studies	
A. Does RHLP medical students’ academic performance equal peers’?^a^ (15)	Quasi-experimental
B. Does RHLP medical students’ choice of Family Medicine equal peers’? (16)	Quasi-experimental
C. Does RHLP graduates’ choice of rural practice equal peers’? (10)	Quasi-experimental
D. Does RHLP participation produce non-physician health professionals? (25)	Retrospective cohort
II. Formative studies	
A. What was the RHLP experience with Black Belt students? (26)	Descriptive cohort
B. What are AA alumni and rural medical educator views of RHLP? (27)	Focus groups
III. Contextual studies	
A. Do counties’ numbers of medical students correlate with life expectancy? (28)	Prevalence study
B. Does institutional commitment correlate with rural physicians? (33)	Prevalence study
C. Do students from small colleges differ from other matriculants? (21)	Prevalence study
D. What RMS characteristics correlate with choice of family practice? (36)	Prevalence study
E. What do rural medical educators advise for new rural medicine programs? (37)	Focus group
F. Can physicians and extension agents cooperate to teach Agromedicine? (40)	Focus group
G. What interest do practicing physicians have in agricultural medicine? (41)	Prevalence study
H. What are rural physicians’ views of long-term community preceptorships? (42)	Focus group
I. What are farmers’ views on medical education needed? (43)	Focus groups
J. What are limited resource AA farmers’ views of farming health and safety? (44)	Interviews
K. How does Industrial Hygiene relate to farmers’ healthcare? (45)	Case report
L. What are the 3-year results of a rural school-based child health program? (46)	Cohort study
M. What health conditions are prominent in a 10-year child health study? (47)	Cohort study
N. What sources of health information do rural households use? (48)	Prevalence study

AA, African American; RMS, Rural Medical Scholars.

a References are numbered as they appear in the reference section.

#### *Evaluation studies* (I) assessed RHLP outcomes

The overall *a priori* evaluation question asked if RHLP produced rural physicians at a rate exceeding SOM’s existing program. We used educational epidemiology (24) with longitudinal designs to pursue 4 subordinate questions as students progressed through RHLP. We addressed the first three questions with a non-randomized intervention study (i.e., quasi-experimental) with multiple controls. The intervention group was RMS that matriculated to SOM (RMS-MD). Contemporary classmates on the main campus (reference group) and non-RMS peers on branch campuses were control groups. Medical school academic performance and choice of family medicine training were intermediate outcomes; rural Alabama family practice was the policy-relevant outcome. Questions, as shown in Table 3, were addressed as soon as enough participants to produce statistically valid results reached the outcome under study.

##### Question I. A

After five classes of RMS (entering medical school 1997–2001) had graduated from medical school (*n* = 47), academic performance was determined by adjusted standardized test scores after preclinical (United States Medical Licensing Examination [USMLE] Step 1) and clinical (USMLE Step 2) coursework and graduation rates. We compared RMS performance to that of their classmates with statistical models that adjusted for sex, MCAT score, and premedical GPA (15).

##### Question I. B

After nine RMS classes (1997–2005) had graduated, RMS’s (high dose RME) selection of family medicine residency was compared with two control groups, non-RMS students at branch campuses (moderate dose) and main campus students (low dose). The logistic regression analysis adjusted for sex, race, MCAT score, and four-year graduation rate (16).

##### Question I. C

When 54 RMS matriculating 1997–2002 had completed residency and entered practice, we addressed uptake of rural Alabama practice among RMS and control groups. Rural practice was defined by RUCA ZIP Codes≥ 4. Logistic regression adjusted for sex, MCAT score, and four-year graduation rate. A geographic analysis mapped home and practice counties of RMS choosing rural practice in Alabama (10).

##### Question I. D

This question addressed the RME pipeline programs’ contribution to the non-physician healthcare workforce. RHLP data from 1993 to 2018, including 642 RHS, 179 RMHS, and 230 RMS participants and the outcomes of 216 health professionals and 70 family physicians, supported a retrospective cohort study. We studied Alabama’s 67 counties to examine the relationship between county participation (i.e., number of student participants) in pipeline programs and number of family physicians gained and other health professionals (e.g., nurses, dentists, pharmacists, and technicians) produced. Linear regression models for each RHLP program assessed the relationship while controlling counties’ rurality, poverty, race, and education level (25).

#### *Formative studies* (II) sought information to improve the RHLP

As a consequence of the geographic distribution of RMS alumni practice sites, we sought to better understand recruitment and medical education experiences of RMS alumni from the Black Belt to inform future adaptations in RME for this region. For Question II. A, we searched the literature to describe the Black Belt and for RME programs addressing similar regions.

Association of American Medical Colleges (AAMC) data showed the distribution of rural AA medical students among all US allopathic medical schools, and we retrospectively tracked 16 AA students’ participation and progress, 1996–2017, in the RMS program to note their completion rate of RMS-MS and RMS-MD (26).

Question II. B led to a focus group study of the RMS program with 10 AA alumni and 7 rural medical educators exploring opinions about adapting RME to regional populations like the Black Belt (27).

#### *Contextual studies* (III) were integral to RHLP development

These studies supported the rationale for differentiated RME and engaged stakeholders from students’ formative communities as partners in support and conduct of programs. These studies evolved organically during program implementation as opportunity generated from program and stakeholders’ interests permitted.

Two studies addressed the rationale for differentiated rural medical education. III. A explored Alabama counties by path analysis (28) testing for an association between number of local medical students and the established relationship of local primary care physicians to population health (29). III. B used linear regression to test for a correlation between medical school commitment to rural medical education and the output of rural physicians (30) using published medical school data (31, 32) and a primary survey to create a 32-item Rural Commitment Index (RCI) (33).

We explored admission characteristics with two studies. III. C employed SOM archival data to examine characteristics and outcomes of matriculants who had attended small Alabama colleges (21). III. D surveyed 64 RMS alumni to explore factors (34, 35) correlated with choice of family practice residency training (36).

Four studies sought information to plan RMS education to address rural concerns that diverge from what is taught traditionally. In III. E, we convened a focus group with 15 members of the National Rural Health Association’s Rural Medical Educators Group to identify key elements to a successful rural medical education program (37). Another focus group (III. F) engaged medical faculty and agricultural extension agents to discuss ways to teach students about agriculture-related health concerns (38, 39) and plan farm field trips (40). We surveyed primary care physicians (III. G) to determine interest in teaching agricultural medicine (41). A qualitative study (III. H) of 19 rural family physicians discussed extending the two-month rural community clerkship to an eight-month longitudinal integrated rural curriculum (42).

We pursued three topics with six studies related to stakeholder interests. The farm community wanted to participate in the development of RME responsive to farm families. Farmers and cooperative extension agricultural agents invited us to explore farmers’ expectations of RME (III. I) through focus group discussions (43). Limited resource AA farmers shared their concerns about agricultural health and safety (III. J) with interviews (44). Two farm workers presented the opportunity for a case-study of pesticide poisoning and its care including Industrial Hygiene (III. K) (45).

Child health was of interest to teachers, health care providers, and parents. We completed a school health program with annual surveys of health, health care, and insurance coverage in a K-12 county school system reporting 3-year (III. L) (46) and 10-year (III. M) (47) results.

The cooperative extension system collaborated with us to survey rural households for their sources of medical information (III. N) (48).

## Synthesis of findings

The RHLP aimed to produce rural physicians in all population groups, however, success was seen only in the white population. Adaptations were community-centric and longitudinal with local students, family medicine focus, and community-based curriculum. Individuals and agencies from rural communities collaborated to nurture students’ rural affinity and ambitions from high school through medical school and to shape the curriculum. The RHLP demonstration included two precollege programs (RHS and RMHS) and the MS-MD rural medicine track (RMS) conducted from the branch campus, except the preclinical medical sciences.

### Evaluation studies (I)

Evaluation of the RMS-MD component confirmed RHLP’s utility. RMS’s academic performance in medical school (USMLE Step 1 and 2 tests and graduation rates) was not statistically different (*p* > 0.05) from the remainder of the class (*n* = 787) when adjusted for gender, MCAT score, and premedical GPA (15). Table 4 shows the RHLP’s effects on choice of family practice training (16) and uptake of rural practice in Alabama (10).

**Table 4:** Family medicine choice and rural practice by Rural Medical Scholars and control groups.

		1997-2005^a^					1997–2002		
		Family medicine choice (16)					Rural practice location (10)		
Campus group	Rural level	*N*	FM%	OR^b^	*P*	*N*	RP%	OR	*P*
Main	Minimal	840	3.9	1	–	649	11.2	1	–
Regional	Moderate	296	18.9	5.8	0.001	182	23.8	2.5	<0.001
RMS	High	84	44.0	15.6	<0.001	54	48.1	6.4	<0.001

FM, Family Medicine; OR, Odds ratio; RMS, Rural Medical Scholars; RP, Rural practice. Data adapted from Wheat et al. (10, 16).

a Dates show the time periods of matriculation to medical school.

b Odds ratios were adjusted for independent variables distributed differently among comparison groups.

Eighty-four RMS that matriculated to medical school, 1997–2005, exceeded 296 branch campus students who in turn exceeded 840 main campus students in selection of family practice residencies (44.0, 18.9, and 3.9%, respectively) by substantial and highly significant rates (RMS OR = 15.6, *p* < 0.001), exhibiting an RME dose–response effect with RMS > branch campus > main campus (16).

Fifty-four RMS (1997–2002 matriculants) compared similarly to 182 branch campus and 649 main campus peers in uptake of rural practice in Alabama: 48.1% (OR 6.4, *p* < 0.001), 23.8% (OR 2.5, *p* < 0.001), and 11.2% (OR 1.0), respectively. Again, a dose–response effect was apparent with RME exposure (10). These results were comparable to the benchmark programs in Minnesota (49), Pennsylvania (50), and Illinois (51). A regional effect appeared among the geographical distribution of RMS family physicians in rural Alabama. Of three RMS physicians from the 17-county Black Belt region, one located in practice there. Of the other RMSs, 24 located among the remaining 38 rural counties, 24 in non-rural locations, and none in the Black Belt (10).

The retrospective cohort study (I. D) of 67 counties’ participation in the RHLP showed a positive relationship between the number of county participants and both family physicians and non-physician health professionals produced. Linear regression models of the three RHLP programs showed the best model for counties’ acquisition of family physicians from the RHLP (R^2^=0.30) included the number of RMS participants per county (b = 0.24, *p* < 0.001); for each four RMS participants a county gained, on average, one family physician. The best model for health professionals produced per county (R^2^=0.31) included the number of RHS per county (b = 0.20, *p* < 0.001), indicating that for each five RHS participants the county produced one health professional. From the RMHS models, a county gained one family physician for each 33 and one health professional for each 7 participants, neither of these results reached statistical significance (*p* > 0.05) (25).

### Formative studies (II)

In response to regional variation in RHLP impact, these studies disclosed conditions of economics, education, demographics, and public resources that distinguished the Black Belt. Table 1 shows Alabama’s population is 27% AA, but 60% in the 17-county Black Belt. Most applicants to RHLP programs from this region were African American (AA). AAMC data showed that, nationwide, less than 0.01 percent of medical school matriculants were rural AA (52) (i.e., six matriculant per year from an estimated six million rural AA). We found no precedents in the literature of medical education designed to prepare southern rural AA to become physicians. For II. A, Table 2 shows that among participants in the RHLP, 30% of RHS and 97% of RMHS were AA, while 7.3% (16 of 220) of RMS were. All 16 AA RMS completed RMS-MS, 12 entered medical school (RMS-MD), and 10 completed medical degrees (26). However, consistent with AAMC data (52), the number of AA RMS (one every two years) was too small for statistical analysis.

The focus group with rural medical educators and minority RMS alumni (II. B) suggested critical factors in the development of physicians for the rural Black Belt region categorized according to an ecological model. Table 5 demonstrates the hypothetical categories of interpersonal relationships, nurturing local community, institutional climate, and supportive policies (27).

**Table 5:** Suggested critical factors for developing rural African American physicians for Alabama.

Ecological level	Suggested factors
Interpersonal relationships	Peers, friends, and community members, especially racially similar, as role models/mentors Counselors, advisors, program directors, and faculty that are trusted and culturally competent
Nurturing community	Family, church, schools, and trusted community members, e.g., cooperative extension agents Local health professionals and health care establishments
Institutional climate	Supportive policies, e.g., financial support, flexibility in admission criteria and curriculum, and assigned advocates Cultural competence among administrators, faculty, staff, and students
Policy support	Recognize rural racial minorities as priority representatives of their underserved populations Reconcile perspectives of institutions and rural minority communities Support holistic approaches to professional education and practice tailored to rural minority communities

Adapted from Wheat et al. (27). Used by permission under CCBY-NC-ND 4.0.

### Contextual studies (III)

These studies served important purposes with RHLP. Two studies supported the rationale for RME implementation. III. A showed that in Alabama the number of medical students from a county correlated positively with the number of primary care physicians (b = 0.37, *p* < 0.001) that, in turn, correlated positively with life expectancy (b = 0.29, *p* = 0.005) in a path analysis that controlled county rurality and poverty. County rurality correlated negatively with the number of medical students (b = −0.24, *p* = 0.043) (28). III. B showed that, nationally, an index of medical schools’ commitment to RME correlated positively (r = 0.52, *p* < 0.001) with graduates in rural primary care in a regression model (R^2^=0.48) that included states’ % rural population (r = 0.41, *p* < 0.001), National Institutes of Health (NIH) research support (r = −0.29, *p* = 0.007), and number of graduates (r = −0.22, *p* = 0.06) (33).

Two studies explored recruitment and admission of rural medical students. III. C suggested that SOM students from small in-state colleges were more diverse than their peers, had a greater struggle completing medical school, and more often practiced in Alabama (5.6% vs. 3.8%). These differences were resolved when adjusted for race, age, premedical science GPA, and MCAT, suggesting that these students contribute equally with their peers to rural practice (21). The survey of 64 RMS alumni (III. D), though too small to adjust for other factors, suggested bivariate correlations between family practice residency and both prematriculation attraction to family practice and commitment to rural and underserved communities (36).

Four studies provided insights for teaching rural medicine. The Rural Medical Educators focus group (III. E) suggested that successful rural medical education program development should include rural-oriented students, curriculums that require training in rural contexts with rural patients, dedicated rural preceptors, program financial security, and program evaluations (37). The focus group with RHLP faculty and agricultural extension agents (III. F) led to an experiential curriculum for agricultural medicine, including field trips to farms that introduced various roles (farmer, extension agent, and local physician) and explored farmers’ concerns and the circumstances in which they lived and worked (40). The survey of primary care physicians (III. G) indicated that potential RME preceptors were interested in agricultural medicine, an interest that correlated with family practice, rural background, and personal experience in agriculture (41). The qualitative study of 19 rural family physicians (III. H) yielded the opinion that a long-term rural clerkship (> 6 months) should include preference for rural students with interest in family medicine and that the roles of preceptor, students, medical school, and community be clarified (42).

Rural Alabama stakeholders catalyzed six studies drawing attention to their interests. In focus group sessions (III. I), farmers’ greatest concern was for physicians who understood their farming culture and could make decisions that matched farmers’ realities (43). From the LRAA farmer interviews (III. J), 6 themes characterized their concerns: limited capital and sources of information, distrust of public institutions and agencies, old unsafe machinery and equipment, a pragmatic resilient attitude, lack of safety training useful on their farms, and personal health conditions (44). The case-study of farm workers with pesticide poisoning (III. K) demonstrated the value of Industrial Hygiene in caring for agricultural workers and the safety of their workspaces (45).

In the rural child health program studies, we found that after 3 years (III. L) uninsurance rates and referrals for dental care decreased and referrals for primary care increased (46). After 10 years (III. M), prevalence of overweight/obesity increased from 17 to 23% with an associated increase in referrals for blood pressure elevation, and medical care utilization was more common among obese students (47).

The rural household survey (III. N) showed that personal physicians and pharmacists were preferred sources of health information. Households with personal computers and the internet used them occasionally to contact their physicians and find additional information (48).

## Discussion

In Alabama, 1993–2017, we found that a comprehensive RME model from the Northeast and Midwest adapted well to rural Alabama, except for the 17-county Black Belt. Adaptations to the model that centralized rural communities in RME development led us to preprofessional pipeline programs and a master’s program in Rural Community Health.

These adaptations supported students’ academic preparations and helped, we hypothesize, to consolidate their rural identity (53) prior to entering medical school in a rural track including admission of rural students, clinical years directed from a regional branch campus, Family Medicine instructors, and community-based rural instruction. Medical education in the community emphasizing family practice, in our opinion, affirmed RMS rural identity and aspirations as professional competence was being achieved (54). Continuous engagement with rural communities and advocates is critically important to affirm students’ aspirations as rural denizens, provide exposures and experiences integrating social and professional ambitions, and, thereby, buttress students’ resolve to complete training for rural service. SOM accepted this premise by institutionalizing the rural track and replicating the RMSP at an additional branch campus (55), thus addressing both the research and rural service missions. Judging the RHLP to be successful, the Alabama Legislature provided continuing state funding, and SOM institutionalized and expanded the RHLP through the University of Alabama System.

The diverse contextual studies that arose during the course of RHLP implementation were vital to adapting program experiences to the mutual interests of medical education and the communities. More than information, these studies consolidated community-program collaborations that addressed specific community interests and generated support for the programs including scholarships, advocacy, and local experiences. This process of community engagement would be expected to provide different results in different contexts leading to differentiation of RME to fit community needs.

The Black Belt benefited from preprofessional RHLP programs but, similar to Native Americans and indigenous populations worldwide (56), had few students enter medical school (57). We attribute much of the success of the RHLP to engagement with rural communities and stakeholders, an engagement that was more difficult to affect and maintain with Black Belt communities whose traditions developed differently from the larger society. RHLP efforts were rooted in majority thought from Willard (3), traditional medical education, and local physicians, administrators, and community members (14). As the limitations of this approach became apparent, we sought information from the literature and from explorations that privileged the voices of Black Belt alumni of the RHLP and citizens. The analysis of qualitative data (27) was sobering—interventions at all ecological levels of human behavior (i.e., interpersonal, institutional, community, and policy) may be necessary to affect and maintain a system of education and care in this socially marginalized region.

Work from the World Health Organization provides a model for building medical education to match findings suggested by the exploratory studies among Black Belt alumni and farmers and informal inquiry with local ministers and elected officials. Boelen’s unity for health concept (58) places the development of medical education among marginalized communities, guided by indigenous as well as professional thought in a multi-sector collaboration. Social accountability operationalized through engagement with the community to be served and additional partners is at the core of this model (59, 60). Adaptations in current policies and institutional traditions, as exemplified by Boelen et al. (61), will require consideration if modern medical education is to accommodate to healthcare needed in these communities and overcome counterproductive aspects of tradition (62). The utility of this approach is suggested through experience in Africa and well chronicled in North America at the Northern Ontario School of Medicine (62–64), where standards of modern medical education and indigenous concepts found common cause to produce doctors serving a large diverse region of Ontario (65).

### Study limitations

The RHLP model was incompletely operationalized yet included three basic components following precepts of comprehensive rural medical education, except preclinical medical sciences were not conducted in a nonurban context. RHLP evaluation design, a non-randomized intervention study with multiple control groups, had strong internal validity. However, generalizability was limited in that the studies represented one southern state. RHLP’s replication of successful rural programs from other regions of the US modifies this limitation, as does RHLP’s successful adoption by another regional campus in Alabama (55). Areas similar to Alabama and benchmark states may find the RHLP experience useful among majority populations.

## Conclusion and recommendations

In conclusion, RHLP applied principles of rural medical education within a framework of community engagement to produce physicians for rural Alabama at multiple times the rate of traditional medical education. This engagement was activated by inclusive program planning and collaborations in studies to inform programs’ emphasis. Information generated helped adapt the RHLP for relevance to much of rural Alabama. However, modifications to accommodate regional distinctions are required for success among Alabama’s Black Belt population.

This compilation of research provides evidence for addressing persistent primary care workforce needs in rural Alabama and perhaps other populations that find their circumstances to be similar. To address the continuing decline in rural physicians (66), the author recommends expansion of differentiated rural medical education programs shown to be successful among dominant rural populations (67), continuation of research to perfect models responsive to diverse rural communities, and formulation of policies to translate this knowledge into common use.

Since the RHLP was institutionalized in 2018 and continues development, we have turned attention to understanding and employing local knowledge from the Black Belt to inform high quality and culturally consonant medical education and care that will be effective in this population (62, 68–72). The current era of intense rural needs and tight-fisted fiscal policy makes this mission interesting and challenging, one that will require the collaboration of multiple sectors, as exemplified by the unity for health model (58), to advance.

## Data Availability

The original contributions presented in the study are included in the article/supplementary material, further inquiries can be directed to the corresponding author.
